# Lower Levels of Blood Zinc Associated with Intradialytic Hypertension in Maintenance Hemodialysis Patients

**DOI:** 10.1007/s12011-020-02385-4

**Published:** 2020-09-15

**Authors:** Yun Liu, Yuanyuan Zheng, Liangtao Wang, Xiaoshi Zhong, Danping Qin, Wenxuan Chen, Rongshao Tan, Yan Liu

**Affiliations:** 1 Department of Nephrology, Guangzhou Red Cross Hospital, Jinan University, No. 396 Tong Fu Zhong Road, Guangzhou, China; 2 Guangzhou Institute of Disease-Oriented Nutritional Research, Guangzhou Red Cross Hospital, Jinan University, No. 396 Tong Fu Zhong Road, Guangzhou, China

**Keywords:** Blood zinc, Intradialytic hypertension, Maintenance hemodialysis, Trace elements, Hypozincemia

## Abstract

Intradialytic hypertension (iHTN) has been related with an increased risk of mortality, with imbalances in trace elements being frequent in maintenance hemodialysis (MHD) patients. The aim of this study was to analyze the relationships between the levels of blood trace elements and iHTN in MHD patients. A total of 144 MHD patients were enrolled in September, 2019 (66 females; 5616 hemodialysis treatments), with a mean age of 64.33 ± 13.39 years and median vintage of 33.50 (16.25–57.50) months. Patients exhibited an average peridialytic systolic blood pressure (SBP) change of − 4.18 ± 20.22 mm Hg in the next 3 months. Thirty-four (23.6%) patients had persistent iHTN (piHTN). These patients were characterized by older age, higher rate of hypozincemia, and modified Charlson comorbidity score, whereas lower blood zinc and hemoglobin, at the time of their recruitment. No significant difference in the levels of other blood trace elements was observed between groups. A general linear mixed (GLM) model showed that with every mg/L point lower mean blood zinc at baseline, the peridialytic SBP change was increased by 4.524 mm Hg (*P* < 0.001). Binary logistic model in modulate of the GLM model revealed that the lower level of blood zinc was associated with piHTN (OR = 0.433, 95 % CI 0.295 to 0.637, *P* < 0.001). Multivariate analysis confirmed both above results. Our study indicated that lower blood zinc was independently associated with piHTN in patients undergoing MHD, but prospective studies with larger population are still needed.

## Introduction

Although hemodialysis (HD) is known to promote a reduction in the levels of blood pressure (BP) in most hypertensive patients, because of the removal of fluids and salt [1], a minority of patients have been shown to regularly exhibit a paradoxical increase either toward or at the end of HD [2]. This phenomenon has been termed intradialytic hypertension (iHTN). There is no consensus regarding its definition, but clinical studies have recently reported iHTN during HD as a rise in the systolic blood pressure (SBP) greater than 10 mm Hg above the level of predialysis SBP [3–5]. Results from cohort studies with a larger sample size showed that 8–13% of HD patients had iHTN [2, 5]. Compared with patients whose SBP were lower during a HD session, patients with iHTN were observed to have an increased risk for hospitalization and mortality [6]. The underlying mechanism of iHTN, which is likely multifactorial, remains to be determined. Studies have shown that older age, shorter dialysis vintage, increased cardiovascular comorbidities, lower body mass index, and lower amount of ultrafiltration during dialysis were the main clinical characteristics of HD patients with iHTN [6]. In addition, activation of the renin–angiotensin–aldosterone system [7], positive sodium balance [8], endothelial dysfunction [4, 9], low intradialytic arterial oxygen saturation [10], and excess extracellular fluid [3] were also considered to be related factors.

Imbalances in trace elements have been associated with the development of cardiovascular disease (CVD), including hypertension [11]. Both hypozincemia and zinc transporter protein abnormalities have been linked to atherosclerosis and microvascular disease [12]. Studies have reported a positive relationship between the levels of serum copper and acute or chronic heart failure [13]. Evidence has also been provided for the important functions of selenium and its selenoproteins in certain CVD, such as Keshan disease and myocardial infarct [14]. Manganese could potentially play a role in controlling blood pressure due to its antioxidative function [15], and significant negative associations between levels of urinary manganese and both systolic and diastolic blood pressure were demonstrated after adjusting for confounding factors [16]. The imbalance of trace elements has been reported to be very common in HD patients [17], and although iHTN belongs to the CVD category associated with the vascular function, rarely studies have focused on the associations between blood or serum trace elements and iHTN. Our study aimed to test the hypothesis that the blood levels of certain trace elements at baseline might be associated with iHTN in HD patients.

## Materials and Methods

### Study Population

Patients undergoing maintenance hemodialysis (MHD) were enrolled in this study in September 2019 at the HD center of the Guangzhou Red Cross Hospital, Jinan University. Inclusion criteria were as follows: (1) HD therapy for more than 3 months, (2) age ≥ 18 years, (3) treated with bicarbonate dialysate and polysulfone membranes, and (4) signed informed consent form. Exclusion criteria were as follows: (1) patients with an expected survival time of less than 3 months or expected to have kidney transplantation within 3 months; (2) patients who had a history of trauma, surgery, or serious infection within the last 3 months; (3) patients who had a confirmed diagnosis of malignancies; (4) patients who refused participating in the study; and (5) patients who were taking trace element supplements or drugs affecting the metabolism of trace elements. Clinical, biochemical, and blood trace elements data were collected following enrollment in the study. Drug usage and dosage were also recorded in the next 3 months. Patients were censored in the event of dialysis modality change, kidney transplantation, transfer to other centers, or recovery of kidney function.

Patients enrolled in the study were subjected to hemodialysis and hemodiafiltration treatments 3 times a week. The machines used were Braun Dialog +(B. Braun Co., Ltd., Melsungen, Germany). A REXEED-15L high-flux polysulfone membrane dialyzer (Asahi Kasei Corp., Tokyo, Japan) with a membrane area of 1.5 m^2^, a blood flow of 180–300 mL/min, a dialysate flow rate of 500 mL/min, and a dialysis time of 4 h, with. Ultra-pure water dialysate was used, and the iron concentrations of dialysate were as follows, respectively: Na^+^ 137.8 mmol/L, K^+^ 2.0 mmol/L, Ca2^+^ 1.5 mmol/L, Mg^2+^ 0.5 mmol/L, Cl^–^ 108.7 mmol/L, CH_3_COO^–^ 4.0 mmol/L, and HCO3^–^ 31.1 mmol/L.

### Measurement of Blood Pressure

The Omron HEM-7133 electronic sphygmomanometer (OMRON Corp., Kyoto, Japan) was used for the measurement of BP in the non-fistula side of the upper arm, and in the right side of the upper arm in patients with catheters. Predialytic BP (preHD BP) was measured after resting for 30 min, whereas postdialytic BP (postHD BP) was measured shortly after the end of dialysis. Accordingly, the peridialytic change in BP was calculated as follows: Peridialytic BP change = postHD BP − preHD BP. All patients were followed for 3 months, and their postHD BPs, postHD BPs, and peridialytic SBP changes were recorded in every HD treatment. Only patients with an average peridialytic SBP increase ≥ 10 mm Hg throughout the entire 3-month observation period were defined as patients with persistent iHTN (piHTN). Sphygmomanometers in our center were regularly calibrated by the manufacturer.

### Demographic, clinical, and laboratorial data

Demographic, clinical, and laboratorial variables were collected following the enrollment of patients in the study.

Demographic variables were as follows: age; gender; predialytic body weight and height; primary kidney disease; smoking; interdialytic weight gain (IDWG); ultrafiltration volume. Drug usage in the next 3 months was also recorded: dosage of recombinant human erythropoietin (rHuEPO); usage of low calcium concentration dialysate (1.5 mmol/L); usage of angiotensin converting enzyme inhibitor (ACEI), angiotensin II receptor blocker (ARB), calcium channel blocker (CCB), β-receptor blocker, α-receptor blocker, and diuretics.

Venous blood samples were collected shortly before the HD session, and then were taken to the clinical laboratory department of our hospital for routine blood and biochemical tests to evaluate the following parameters: red blood cell (RBC), hemoglobin (Hb), hematocrit (Hct),serum creatinine (SCr), serum urea nitrogen (BUN), serum potassium ion (K^+^), serum sodium ion (Na^+^), serum magnesium ion (Mg^2+^), serum calcium ion (Ca^2+^), serum phosphorus (P), parathyroid hormone (PTH), and albumin (ALB). The weekly total Kt/V was measured using the methods described by Gotch et al. [18].

### Blood Trace Element Test

#### Sample Preparation

Venous blood samples before dialysis treatment were collected at the time of the enrollment of patients in the study. After thorough mixing of the blood sample, 100 μL of it was taken to make a 1:19 dilution with diluents containing 0.1 % nitric acid (stock solution 65%, Sigma-Aldrich Co., Ltd., St. Louis, MO, USA) and 0.1% triton (stock solution X-100, 1.01 g/cm^3^, Sigma-Aldrich Co., Ltd., St. Louis, MO, USA) for further use.

#### Quantification of Trace Elements and Standard Curves

Levels of blood trace elements (zinc, manganese, copper, and selenium) were measured using an inductively coupled plasma mass spectrometer (ICP-MS, Agilent 7900, Agilent Technologies Inc., CA, USA). Instrument parameters were as follows: forward power 1550 W; carry gas (Argon) 1.0 L/min; aux gas (Argon) 1.0 L/min; plasma gas (Argon) 15.0 L/min; S/C temperature 2 °C; uptake speed 0.2 r/s; sample depth 6–8 mm; uptake time 30 s; acquisition time 0.1 s; repetition 2; cell entrance − 40 V; cell exit − 60 V; deflect 0 V; plate bias − 60 V; octP RF 180 V; octP bias − 18 V; QP bias − 15 V: gas flow (He) 4.0–5.0 mL/min. All selected trace elements were measured under optimum analytical conditions, as shown in Table 1. The tuning solution used for application (10.0 μg/L) contained lithium/cobalt/cerium/yttrium/thallium elements, while the internal standard solution used for application (20.0 μg/L) contained germanium/rhodium/indium/lutetium elements.

**Table 1:** Quantification of trace elements and standard curves

	STD0	STD1	STD2	STD3	STD4	STD5	LOD	LOQ	Standard solution
Manganese (μg/L)	0.0	5.00	10.0	20.0	40.0	80.0	1.00	3.00	1000 μg/mL
Copper (μg/L)	0.0	60	300	600	3000	6000	5.00	15.0	1000 μg/mL
Zinc (mg/L)	0.0	0.200	1.00	2.00	10.0	20.0	0.100	0.300	10.00 mg/mL
Selenium (μg/L)	0.0	100	200	400	800		2.00	6.00	1000 μg/mL

*LOD*, limit of detection; *LOQ*, limit of quantification; *STD*, standard

#### Modified Comorbidity Index

Modified Charlson comorbidity (CCI), which has been widely used in hemodialysis patients, was employed to assess the comorbidities of patients [19] at the time of their enrollment in the study.

#### Statistical Analysis

Descriptive statistics comprised mean ± standard deviation for continuous variables with normal distribution, median (25–75% interquartile range) for data with a skewed distribution, and percentages for categorical variables, respectively. Patients were stratified based on average peridialytic SBP changes recorded during the following 3-month observation period in the piHTN group (average peridialytic SBP change ≥ 10 mm Hg) and the non-piHTN group (average peridialytic SBP change ˂ 10 mm Hg). Differences between the 2 groups were evaluated using the Student’s *t* test or Mann–Whitney test or *χ*2 test, as appropriate. Both general linear mixed (GLM) and binary logistic regression models in the GLM module of SPSS were performed with univariate and multivariate analysis to determine the variables significantly affecting peridialytic SBP change and piHTN respectively. Repeated-measured data of blood pressure–related variables were applied into the GLM model. Confounding factors were selected based on their documented or hypothesized association with exposure and outcome. The SPSS version 24.0 for Windows (IBM Corp, Armonk, NY, USA) was used for statistical analysis. A *P* value < 0.05 was considered to be statistically significant.

## Results

### Characteristics of Study Population

A total of 144 MHD patients were included in the study in September, 2019. Patients had a mean age of 64.33 ± 13.39 years, median vintage of 33.50 (16.25–57.50) months, with 66 being females (45.8%). The primary cause of end-stage renal disease was diabetic nephropathy (*n* = 63, 43.8%), followed by hypertension (*n* = 25, 17.4%), and chronic glomerulonephritis (*n* = 16, 11.1%). Detailed characteristics of study population are provided in Table 2. After 3 months of follow-up, no patient had dropped out and all patients had completed 5616 HD treatments in total.

**Table 2:** Clinical characteristics of the total study population and after stratification into groups following their enrollment in the study

Variables	Total	Non-piHTN group (*n* = 110)	piHTN group (*n* = 34)	*P* value
Patients, *N* (%)	144	75 (52.1%)	34 (23.6%)	
Demographics				
Age, years	64.33 ± 13.39	63.3 ± 14.05	67.68 ± 10.50	*0.045*
Gender (female, %)	66 (45.8%)	51 (46.4%)	15 (44.1%)	0.846
Vintage, months	33.50 (16.25–57.50)	33.58 (15.57–59.67)	35.00 (18.75–53.75)	0.715
Diabetic (*N*, %)	63 (43.8%)	51 (46.4%)	12 (35.3%)	0.324
Modified CCI	3.00 (2.00–4.00)	3.00 (2.00–3.00)	4.00 (2.75–5.00)	*< 0.001*
Smoke (*N*, %)	35 (24.3%)	26 (23.6%)	8 (23.5%)	0.904
BMI (kg/m^2^)	22.37 ± 4.72	22.27 ± 5.11	22.71 ± 3.17	0.635
Clinical parameters at baseline				
Ultrafiltration volume (mL)	2.42 ± 0.97	2.45 ± 0.93	2.32 ± 1.09	0.487
IDWG (kg)	2.03 ± 1.03	2.05 ± 1.05	1.97 ± 0.98	0.707
PreHD SBP (mm Hg)	143.47 ± 18.79	144.69 ± 19.54	139.53 ± 15.75	0.120
PostHD SBP (mm Hg)	145.32 ± 22.96	137.91 ± 18.95	169.29 ± 17.98	*< 0.001*
PreHD DBP (mm Hg)	72.15 ± 11.67	73.14 ± 12.01	68.94 ± 9.98	0.066
PostHD DBP (mm Hg)	71.29 ± 8.51	69.92 ± 8.14	75.71 ± 8.28	*< 0.001*
Peridalytic SBP^a^ change (mm Hg)	1.85 ± 19.24	− 6.78 ± 10.57	29.76 ± 13.53	*< 0.001*
Peridalytic DBP^b^ change (mm Hg)	− 0.86 ± 11.34	− 3.21 ± 9.88	6.76 ± 12.50	*< 0.001*
Drugs in the next 3 months				
ACEI (*N*, %)	17 (11.8%)	13 (11.8%)	4 (11.8%)	1.000
ARB (*N*, %)	72 (50.0%)	56 (50.9%)	16 (47.1%)	0.845
CCB (*N*, %)	109 (75.7%)	85 (77.3%)	24 (70.6%)	0.494
β-Blocker (*N*, %)	83 (57.6%)	60 (54.5%)	23 (67.6%)	0.234
α-Blocker (*N*, %)	54 (37.5%)	40 (36.4%)	14 (41.2%)	0.686
Diuretic (*N*, %)	64 (44.4%)	48 (43.6%)	16 (47.1%)	0.844
rHuEPO dosage (U/kg per week)	655.87 (184.03–756.72)	655.87 (154.65–756.72)	655.87 (349.61–756.72)	0.756
Low calcium concentration dialysate (*N*, %)	14 (9.7%)	10 (12%)	4 (11.8%)	0.741

^a^Peridalytic SBP = postHD SBP − preHD SBP

^b^Peridalytic DBP = postHD DBP − preHD DBP

### Peridialytic SBP Change and Levels of Trace Elements

**Fig. 1:**
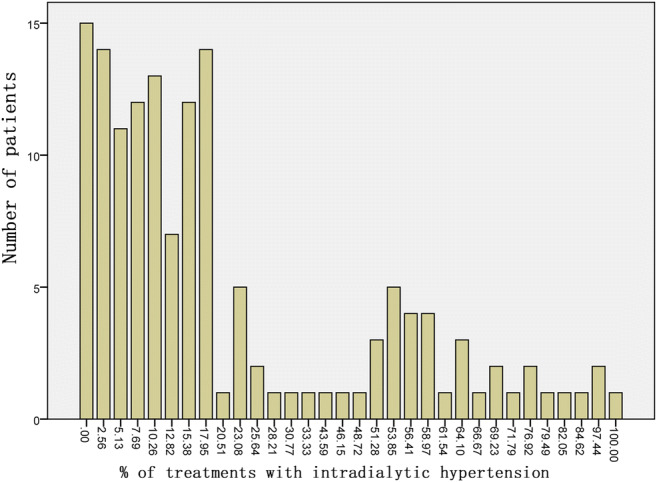
Histogram of percentage of HD sessions with iHTN. Numbers of patient-level HD treatments used as denominators. iHTN was defined as a peridialytic SBP change ≥ 10 mm Hg

At the time of their enrollment in the study, the mean preHD, postHD SBP, and peridialytic SBP change values of patients were 143.47 ± 18.79, 143.47 ± 18.79, and 1.85 ± 19.24 mm Hg, respectively. In the next 3 months, the mean peridialytic SBP change of all patients was shown to be − 4.18 ± 20.22 mm Hg. Figure 1 displays the frequency of iHTN. On average, all patients exhibited iHTN in 14.10 (5.13–32.69) of their treatments.

The mean level of blood zinc in these patients was 5.46 ± 1.38 mg/L, with a normal distribution. The median levels of blood copper, selenium, and manganese were 835.25 (752.38–962.15) μg/L, 103.15 (91.71–112.92) μg/L, and 13.55 (9.93–17.48) μg/L respectively; all with abnormal distributions. Following the recently published prospective study with a large population of MHD patients [20], all cutoffs of trace elements analyzed by ICP-MS in our study were according to those set by Cesbron et al. [21]. According to these cutoffs, a total of 30 patients were identified with hypocupremia, 2 with hypercupremia, 29 with hypozincemia, 18 with hyperzincemia, 10 with hypomanganesemia, 75 with hypermanganesemia, 17 with hyposelenemia, and 6 with hyperselenemia (Table 3).

**Table 3:** Laboratory parameters of the total study population and after stratification into groups following enrollment in the study

Variables	Total	Non-piHTN group (*n* = 110)	piHTN group (*n* = 34)	*P* value
PreHD				
RBC (×10^9^/L)	3.54 ± 0.78	3.69 ± 0.75	3.03 ± 0.66	*< 0.001*
Hb (g/L)	99.83 ± 19.69	102.95 ± 18.47	89.74 ± 20.36	*0.001*
Hct (%)	0.31 ± 0.06	0.32 ± 0.06	0.27 ± 0.06	*< 0.001*
BUN (mmol/L)	20.72 ± 5.60	20.85 ± 5.43	20.29 ± 6.14	0.610
SCr (μmol/L )	765.5 (615.00–901.25)	790.5 (602.75–920.00)	729.50 (650.25–859.00)	0.482
K (mmol/L)	4.31 ± 0.83	4.35 ± 0.85	4.18 ± 0.75	0.295
Na (mmol/L)	137.59 ± 3.66	137.52 ± 3.60	137.82 ± 3.86	0.672
Mg (mmol/L)	1.14 ± 0.16	1.15 ± 0.17	1.11 ± 0.13	0.206
Ca (mmol/L)	2.26 ± 0.19	2.26 ± 0.19	2.26 ± 0.20	0.970
P (mmol/L)	1.85 ± 0.61	1.89 ± 0.65	1.73 ± 0.45	0.101
Ca × P	52.10 ± 18.36	53.14 ± 19.31	48.72 ± 14.62	0.160
PTH (pmol/L)	24.03 (15.13–39.85)	23.26 (14.08–40.46)	27.31 (16.50–37.49)	0.490
ALB (g/L)	36.02 ± 4.02	36.06 ± 4.09	35.89 ± 3.85	0.828
Kt/v	1.28 ± 0.27	1.29 ± 0.28	1.28 ± 0.23	0.812
Blood copper (μg/L)	835.25 (752.38–962.15)	831.65 (753.15–960.25)	844.35 (745.90–999.58)	0.290
Blood copper level < 743 μg/L	30 (20.8%)	22 (20%)	8 (23.5%)	0.637
Blood copper level > 1513 μg/L	2 (1.4%)	2 (1.8%)	0 (0%)	1.000
Blood zinc (mg/L)	5.46 ± 1.38	5.74 ± 1.32	4.52 ± 1.10	*< 0.001*
Blood zinc level < 4.220 mg/L	29 (20.1%)	14 (12.7%)	15 (44.1%)	*< 0.001*
Blood zinc level > 7.198	18 (12.5%)	17 (15.5%)	1 (2.9%)	0.073
Blood manganese (μg/L)	13.55 (9.93–17.48)	14.4 (9.80–18.00)	12.15 (10.65–15.60)	0.383
Blood manganese < 5.9 μg/L	10 (6.9%)	6 (5.5%)	4 (11.7%)	0.247
Blood manganese > 13.3 μg/L	75 (52.1%)	61 (55.5%)	14 (41.2%)	0.171
Blood selenium (μg/L)	103.15 (91.71–112.92)	104.00 (93.89–113.19)	96.81 (87.73–108.48)	0.281
Blood selenium < 85 μg/L	17 (11.8%)	14 (12.7%)	3 (8.8%)	0.763
Blood selenium > 142 μg/L	6 (4.2%)	4 (3.6%)	2 (5.9%)	0.627

### Comparison between piHTN and Non-piHTN Patients

Thirty-four (23.6 %) patients were identified with having piHTN. A comparison between the 2 groups at the time of their enrollment in the study is displayed in Tables 2 and 3. Peridialytic SBP was shown to be increased ≥ 10 mm Hg in 63.3% and 18.8% of treatments in the piHTN and non-piHTN groups, respectively. Patients with piHTN were more likely to be of older age and have higher modified CCI or a higher rate of hypozincemia, whereas lower predialytic Hb, RBC, Hct, and level of blood zinc (all *P* < 0.05). There was no significant difference observed in the levels of other blood trace elements (copper, lead, manganese, and selenium), or other clinical or laboratory markers between the 2 groups at the time of enrollment in the study, or throughout the use of antihypertension drugs, or low calcium concentration dialysate, or dosage of rHuEPO, in the next 3 months.

### Associations between Level of Blood Zinc and piHTN

Table 4 displays the results obtained from GLM models. Unadjusted analysis showed that with every mg/L point lower mean blood zinc, the peridialytic SBP change was increased by 4.524 mm Hg. After adjustment for age, preHD SBP, rHuEPO dosage, and modified CCI in model 2 or an adjustment of model 2 for gender and vintage (model 3), or after adjustment for age, BMI, smoking, gender, and vintage in model 4 or an adjustment of age, preHD SBP, rHuEPO dosage, CCI, BMI, and smoking (model 5), these findings were also corroborated. Furthermore, after adjusting for the abovementioned confounding factors, binary logistic regression in the GLM module of the obtained SPSS results showed that the lower levels of blood zinc at baseline or hypozincemia (defined by a level of blood zinc < 4.220 mg/L) were independently associated with piHTN, respectively (Table 5). No association was observed between neither of the other trace elements and piHTN, nor between other trace elements and peridialytic SBP change in univariate or multivariate analysis.

**Table 4:** Results of GLM models relating indicators of blood zinc (independent variables) with peridialytic SBP change (dependent variable)

	Model 1	Model 2	Model 3	Model 4	Model 5
Blood zinc level	− 4.524 (− 6.719 to − 2.329)	− 3.860 (− 5.977 to − 1.744)	− 3.785 (− 5.891 to − 1.678)	− 4.685 (− 6.844 to − 2.526)	− 3.941 (− 6.072 to − 1.809)
	< 0.001	< 0.001	0.001	< 0.001	< 0.001
Blood zinc level < 4.220 mg/L	− 10.381 (− 18.122 to − 2.641)			− 9.634 (− 17.332 to − 1.937)	
	0.009	0.119	0.175	0.015	0.132

Model 1: Unadjusted

Model 2: Adjusted for age, preHD SBP, rHuEPO dosage, and modified CCI

Model 3: Adjusted for age, preHD SBP, rHuEPO dosage, modified CCI, gender, and vintage

Model 4: Adjusted for age, BMI, smoking, gender, and vintage

Model 5: Adjusted for age, preHD SBP, rHuEPO dosage, modified CCI, BMI, and smoking

**Table 5:** Association between blood zinc and piHTN (by binary logistic regression in GLM module of SPSS)

	Model 1	Model 2	Model 3	Model 4	Model 5
Blood zinc level	0.433 (0.295 to 0.637)	0.470 (0.309 to 0.741)	0.477 (0.314 to 0.725)	0.422 (0.273 to 0.654)	0.464 (0.291to 0.744)
	<0.001	< 0.001	0.001	< 0.001	0.002
Blood zinc level < 4.220 mg/L	5.414 (2.248 to 13.039)	3.595 (1.361 to 9.495)	3.467 (1.294 to 9.289)	5.366 (2.125 to 13.558)	3.880 (1.372 to 10.968)
	< 0.001	0.01	0.013	< 0.001	0.011

Model 1: Unadjusted

Model 2: Adjusted for age, preHD SBP, rHuEPO EPO dosage, and modified CCI

Model 3: Adjusted for age, preHD SBP, rHuEPO dosage, modified CCI, gender, and vintage

Model 4: Adjusted for age, BMI, smoking, gender, and vintage

Model 5: Adjusted for age, preHD SBP, rHuEPO dosage, modified CCI, BMI, and smoking

## Discussion

After adjusting for confounding factors, our research showed for the first time an inverse association between the level of blood zinc and peridialytic SBP change, with lower blood zinc or hypozincemia being independently associated with piHTN in MHD patients.

As an important trace element and micronutrient, zinc has important structural, enzymatic, and regulatory functions, and is essential for the maintenance of a healthy body. Meta-analysis revealed that compared with healthy controls, lower levels of blood zinc or serum zinc were observed in MHD patients [17]; however, in the present study, the mean concentration of blood zinc was 5.46 ± 1.38 mg/L, similar with that reported in the general population with normal renal function [21]. Researchers have also found that 5.1–78% of MHD patients were hypozincemic [22–24], and this was also shown in our study where by using the cutoff by Cesbron et al. [21], we identified 29 patients (20.1%) with hypozincemia (blood zinc < 4.220 mg/L). Perhaps because of not only the use of ACE inhibitors or angiotensin 2 receptor antagonists or thiazide diuretics promoting zinc excretion with urine [25] but also because of decreased intake of zinc in diet, defective absorption of zinc in the intestine [26], and zinc removal in dialysate during HD, MHD patients with lower levels of blood zinc were more likely to be zinc deficient [27]. The rate of hypozincemia was much higher in the piHTN than in other groups (44.1 %), with this group also exhibiting the lower level of blood zinc (Table 3).

Hypozincemia might indicate zinc deficiency in MHD patients, but as the possible mechanisms of the lower levels of blood zinc or hypozincemia were independently associated with piHTN, this complicated the result. First, zinc deficiency has been reported to promote the activation of the hypoxia-inducible factor-1 (HIF-1)/ET-1 pathway, resulting in increased secretion of ET-1 and migration of endothelial cells [28]. Researchers noticed that the availability and function of endothelial NO were likely to be compromised if endothelial zinc stores became depleted in conditions of zinc deficiency [12], while interestingly, clinical studies showed that patients with iHTN had a significant decrease of the NO/ET-1 balance at the end of HD compared with control patients [9], potentially suggesting that zinc deficiency might play an important role in iHTN via the decreased NO/ET-1 balance. Second, through directly protecting easily oxidized groups, such as sulfhydryl and producing some other ultimate long-term antioxidants, such as metallothionein, zinc has been demonstrated to be involved in antioxidative actions. Furthermore, a meta-analysis showed that zinc supplementation could increase the levels of blood or serum zinc and lead to an anti-inflammatory and antioxidative effect in MHD patients [29]. As inflammation and oxidative stress have emerged as novel and major contributors to accelerated atherosclerosis [30], and atherosclerosis was considered to be one of the factors for iHTN [2], we therefore speculated that there might be some indirect connection between the lower levels of blood zinc and iHTN. Lastly, lower levels of blood zinc have been related to lower rHuEPO response and more dosage of rHuEPO for the treatment of anemia in MHD patients. Accordingly, oral zinc supplementation was shown to result in reduced erythropoietin responsiveness index [31], whereas intravenous rHuEPO caused an increase in the mean arterial pressure with an increase in ET-1 [32], which was related to iHTN. We also noticed that there were many studies focusing on the effects of zinc supplementation on MHD patients, but there was no study observing the relationship between zinc supplementation and iHTN.

Our study also reported that the piHTN group was characterized by older age, and higher CCI, whereas lower predialytic Hb, which were also corroborated in other studies with large and diverse populations [3, 5, 10, 33]. We observed that iHTN occurred in 90% of patients at least once in the 3 months of our follow-up, in accordance with other similar studies [10, 34]. However, our patients showed a higher prevalence of piHTN than that reported in previous studies [2, 5]. This could be explained on the basis that our patients were older (mean age 64.33 ± 13.39 years) than patients recruited in those studies [2, 3, 5, 10, 33], and patients with older age might be more likely to have piHTN [5].

Excess of potentially toxic trace elements and deficiency of essential trace elements are known to be common conditions in HD patients, and available data have suggested that levels of copper were higher, whereas levels of selenium, zinc, and manganese were lower in hemodialysis patients compared with controls [17]. Researchers observed that in HD patients, the decreased levels of serum selenium were correlated with diminished coronary flow reserve [35], and a higher ratio of serum copper/serum zinc implied increased cardiovascular risk [36]. However, we did not observe any significant differences in the levels of other blood trace elements (copper, manganese, selenium) between groups, nor any associations between any blood trace elements and piHTN or peridialytic SBP change in our study.

To a certain extent, the results of our study are strengthened because of the 3-month follow-up in assessing and defining piHTN. But admittedly, there have been a few limitations in our study. First, as this was a cross-sectional and observational study, and although we could note a possible protective effect of sufficient zinc in iHTN, no conclusions towards causation could be drawn. Second, multivariable models were performed after adjusting for many factors, but this study included a relatively small sample size. Therefore, our results should be cautiously interpreted due to a certain degree of unknown bias. Third, we conducted repeated measurements on blood pressure, but we only once tested the levels of blood zinc at the time of the enrollment of patients in the study. Fourth, we did not collect information on the source water used for HD or on the urine content of trace elements, residual kidney function, dietary intake, or extent of albuminuria, all of which could be associated with the level of blood zinc. Lastly, data related to endothelial dysfunction, such as plasma ET-1 and NO, were not recorded, and we acknowledge that these data might have been helpful in analyzing the relationship between the level of blood zinc and endothelial function.

## Conclusions

Not much attention has been paid on the relationship between trace elements and iHTN, and hence, this is the first study demonstrating an association between lower levels of blood zinc and iHTN, after adjustment for confounding factors. We speculated that the underlying cause might be the negative effects of zinc deficiency on the vascular and endothelial function of MHD patients. Therefore, more attention should be paid to the levels of blood zinc for the management of iHTN in MHD patients, as iHTN has been associated with increased risk for hospitalizations and mortality. Prospective studies with larger sample size and with zinc supplementation are needed in the future in order to clarify the exact role of zinc in iHTN.
